# Association of School Education With Eyesight Among Children and Adolescents

**DOI:** 10.1001/jamanetworkopen.2022.9545

**Published:** 2022-04-29

**Authors:** Chunfeng Zhang, Ling Li, Catherine Jan, Xiang Li, Jia Qu

**Affiliations:** 1 National School of Development, Peking University, Beijing, China; 2 Lost Child’s Vision Project, Sydney, Australia; 3 School of Ophthalmology and Optometry, Eye Hospital, Wenzhou Medical University, Zhejiang, China

## Abstract

**Importance:**

Myopia in youths is an increasingly common global problem, associated with significant social and economic burden worldwide.

**Objectives:**

To explore the association of school education with myopia and investigate its scale, controlling confounding factors, such as age and socioeconomic status.

**Design, Setting, and Participants:**

This cross-sectional study was conducted from July 2019 to December 2020 using regression discontinuity (RD) based on the Compulsory Education Law to examine the complex association of school education with myopia. Participants were youths from grades 1 to 12 before tertiary or university studies in China, including individuals from 815 primary schools, 373 junior high schools, and 145 senior high schools. Data were analyzed from February through June 2021.

**Exposures:**

Participants born earlier in a year (ie, January to August) were assigned 1 more year of education and were considered to be in the treated group, while participants born September to December were considered to be in the control group.

**Main Outcomes and Measures:**

Differences in mean spherical equivalent refractive error (MSE) and uncorrected visual acuity (UVA) between treated and control groups were calculated.

**Results:**

Among a mean (SD) of 812 979 (55 953) participants across 5 surveys survey (mean SD 44.9% [0.5%] female participants; mean [SD] age, 11.19 [2.60] years), the mean (SD) MSE was −1.30 (2.93) diopters and mean (SD) UVA was 4.77 (0.34) points. Every additional year at school was associated with an decrease in MSE of −0.17 diopters/y (95% CI, −0.22 to −0.11 diopters/y) and a decrease in UVA of −0.03 points/y (95% CI, −0.04 to −0.02 points/y), which accounts for nearly 66.4% (95% CI, 85.9% to 43.0%) and 78.9% (95% CI, 102.6% to 55.3%) of changes of MSE and UVA, respectively, among all factors for youths. The greatest MSE shifts occurred in grades 3 and 7 (eg, for the June 2019 census, the RD was −0.26 diopters/y [95% CI, −0.33 to −0.20 diopters/y] for grade 3 and −0.41 diopters/y [95% CI, −0.66 to −0.15 diopters/y] for grade 7).

**Conclusions and Relevance:**

These findings suggest that receiving more years of education, instead of age, may be a risk factor associated with myopia progression in youths.

## Key Points

**Question:**

Are more years of school education associated with increased rates of myopia?

**Findings:**

This cross-sectional study of a mean of 812 979 youths across 5 surveys in China found that every additional year of school education was associated with a decrease in mean spherical equivalent refractive error (MSE) and a decrease in uncorrected visual acuity. The greatest MSE shifts occurred in grades 3 and 7.

**Meaning:**

This study found that each year of education was associated with an increase in the risk of myopia.

## Teaser

This cross-sectional study investigates the association of 1 additional year of education with mean spherical equivalent refractive error and uncorrected visual acuity among youths in China.

## Introduction

Poor vision poses significant social and economic cost.^1^ Of children with poor vision worldwide, approximately half live in China.^2^ Recent studies found that 80% of all school graduates in China had visual impairment, and among them, nearly all individuals had myopia.^3^ The prevalence of myopia is also increasing in other parts of the world.^4,5,6,7,8^ Although this is usually a benign condition, untreated severe myopia may be associated with increased risk of potentially blinding pathologies, such as cataract,^9^ glaucoma,^10^ and pathological myopia.^11^ Thus, it is important to understand the risk factors associated with myopia onset and progression so that viable treatment and management plans may be carried out. Many countries have recently adopted policies to curb the growing burden of myopia.^12^ A 2018 policy from the Chinese State Council targeted a reduction of myopia prevalence among schoolchildren by more than 0.5% per year from 2018 to 2023 in all provinces and a figure below 70.0% among high school students by 2030; however, these targets have not been achieved thus far.^12^

Known risk factors associated with myopia include less time outdoors,^13^ increased near work (ie, close-up use of the eyes, such as close-up reading of texts),^14^ education,^15^ parental myopia,^16^ and age. Education has been consistently shown as a factor associated with one of the largest increases in risk of myopia.^15^ Myopia prevalence is particularly high in countries like China that have adopted an intense education scheme at primary and secondary schools.^17,18^ However, the association of education with myopia is often confounded with age. To untangle this complex association, we used a regression discontinuity (RD) design taking advantage of China’s Compulsory Education Law to conduct a study on youths born in the same year with length of education as treatment. To our knowledge, this study provides the largest sample known to date (nearly 1 million) for the study of myopia in youths from all school grades.

## Methods

This cross-sectional study received ethics approval from the ethics committee of the Eye Hospital of Wenzhou Medical University and complied with the Declaration of Helsinki. Written consent was not obtained from each participant because all data were used anonymously. This is also approved by the ethics committee of the Eye Hospital of Wenzhou Medical University. We followed the Strengthening the Reporting of Observational Studies in Epidemiology (STROBE) reporting guideline.

### Study Design and Data Sources

China’s Compulsory Education Law requires individuals aged 6 years and older to receive education until they finish junior high school, which is referred to as 9-year compulsory education. In practice, children who are born before September 1 would be required to go to primary school in the year they turn age 6 years, while those who are born on or after September 1 start school in the year they turn age 7 years because the school semester begins on September 1 in China. Thus, among all children born in a given year (eg, in 2009), those born earlier in a year (ie, January to August) are assigned 1 more year of education naturally compared with children born later in that year (ie, September to December).

Thus, we regarded individuals born before September 1 as members of our treatment group in the context of this controlled study, while individuals born later were considered members of the control group. Our basic study design was to compare mean spherical equivalent refractive error (MSE) and uncorrected visual acuity (UVA) of the treatment group with that of the control group. The bandwidth of birth date narrowed to a small period around September 1 to minimize differences between treated and control groups through the automatic selection process suggested by Calonico, Cattaneo, and Titiunik.^19^

The main data used in this article were from the censuses of myopia among all eligible youths in Wenzhou, China, where prompt and frequent censuses were launched before those in the whole country after China’s 2018 policy, covering all students before tertiary or university studies. Until March 2021, 5 waves of censuses that launched in June 2019, September 2019, December 2019, June 2020, and December 2020 were completed. There was no census in September 2020 because of COVID-19. In total, 815 primary schools, 373 junior high schools, and 145 senior high schools were included.

Visual acuity testing and noncycloplegic autorefraction were carried out for both eyes of all participants by certified opticians who had undergone standardized training. The electronic logarithmic visual chart Goldeye CM-1900C and autorefractometer Goldeye RM-9000 were used in all schools. Spherical equivalent refractive error was used for analysis and was calculated for each eye by the formula spherical equivalent = spherical power + 0.5 × cylindrical power, as a major index of visual ability. The mean of left and right spherical equivalent refractive error was calculated and used as MSE in analyses. When data from 1 eye were unavailable, MSE was taken from the other eye. UVA was measured using logarithmic visual acuity standard and then the mean found across both eyes. MSE and the UVA were used as our dependent variables.

### Study Population

To investigate the association of school education with eyesight among youths, we limited our sample to youths aged 6 to 18 years because participants outside of this range have a limited sample size. Furthermore, we excluded individuals who lacked comparable peers born in the same year in our sample given that they did not have corresponding treatment or control groups. The Ministry of Education of China strengthened the implementation of China’s Compulsory Education Law beginning in 2010.^20^ Therefore, we further excluded individuals born before 2004 so that all individuals included had turned age 6 years by 2010. In total, 740 201 participants, 820 869 participants, 796 848 participants, 810 935 participants, and 896 044 participants were valid for our analysis for 5 cross-sectional censuses, respectively. The process of sample restriction is reported in detail in eTable 1 and eFigure 1 in the Supplement.

### Statistical Analysis

We used the RD method to control for potential unobserved time-invariant and time-variant individual factors.^21^ Time-invariant factors were excluded by comparing students born earlier in a year with those born later. For time-variant factors, influence at year level and higher levels were collapsed to each birth month and therefore offset by themselves. If we assumed any confounding factors were not correlated with birth date, or more specifically to birth month of August or September, the estimation results represented the association of 1 more year spent on school education with student eyesight. This was tested afterward in validity testing.

The key variable of interest is *D_i_* in the regression model (eMethods in the Supplement). Its coefficient, δ, captures the association of 1 more year spent in school education with eyesight, or the local mean treatment outcome because the estimation is narrowed to a small bandwidth around the birth date August 31. The analytical solution for the outcome is also provided in eMethods in the Supplement. We used *t* test is used to evaluate 2-sided *P* values. The level of statistical significance was demonstrated as *P* < .01, *P* < .05, and *P* < 0.10, with 3 different levels of testing for each regression result or coefficient, for results reported in the Supplement. We used *P* < .001 as the level of statistical significance for all results reported in the study. Data were analyzed using Stata statistical software version 14.1 (StataCorp) from February through June 2021.

## Results

### Summary Statistics

**Figure 1.**
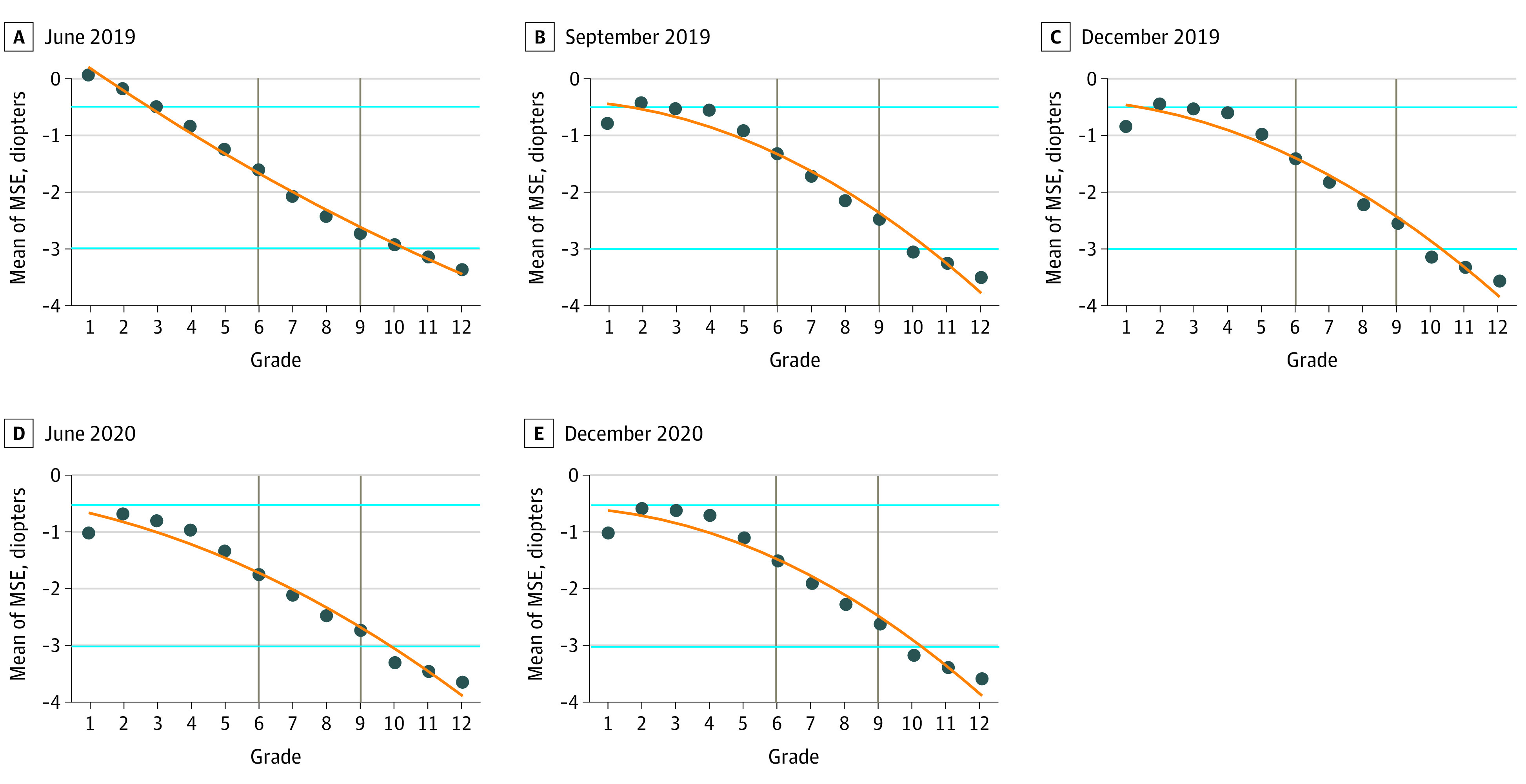
Mean Spherical Equivalent Refractive Error (MSE) by Birth Year and Grade for Each Census Blue lines indicate cutoff values of myopia diagnosis; gray lines, grades 6 and 9. Arithmetic mean of MSE is calculated by grade.

A total of 4 064 897 participants for 5 censuses, with a mean (SD) of 812 979 (55 953) participants per census (mean SD 44.9% [0.5%] female participants; mean [SD] age, 11.19 [2.60] years) were included in this study. Mean (SD) MSE and UVA were −1.30 (2.93) diopters and mean (SD) UVA was 4.77 (0.34) points, respectively. Detailed information on spherical power (mean [SD], −0.96 [2.93] diopters), cylindrical power (mean [SD], −0.69 [1.11] diopters), and associated demographic variables is reported in eTable 2 in the Supplement. Figure 1 presents the mean MSE of all students by school grade; mean UVA is presented in eFigure 2 in the Supplement. Eyesight of youths decreased with year of education. Students acquired low myopia (ie, mean MSE less than −0.5 diopters) when in grade 2 and moderate myopia (ie, MSE less than −3.0 diopters) in grade 10. Moreover, low poor vision (ie, mean UVA <5.0 points) began in grade 1, moderate poor vision (ie, mean UVA <4.8 points) in grade 5, and severe poor vision (ie, mean UVA <4.5 points) in grade 10.

### Results From Econometric Analysis

In this section, we present validity testing for the RD design. The assumption of RD is that confounding factors were not associated with one’s birth month of August or September. Therefore, factors that may be correlated with it, including age, sex, intensive school, and parents’ manipulation of birth time or year, were tested.

#### Testing Validity of RD Design

Students born before September had worse MSE than students born in September and afterward (eg, mean [SD] MSE, −1.40 [6.18] diopters vs −1.09 [4.00] diopters in the June 2019 census) and UVA (eg, mean [SD] UVA, 4.75 [0.34] points vs. 4.80 [0.33] points in the June 2019 census), and a sharp jump at the cutoff of August 31 (eFigure 3 and eFigure 4 in the Supplement). This sharp jump was associated with factors that are associated with birth month and possibly with 1 year of education at school if other risk factors associated with myopia were found to not be associated with birth month.

Parents may manipulate students’ birth dates (eg, by manipulating the reported date or taking family planning steps to have children at specific times) to give children a head start; however, in eFigure 5 in the Supplement, there was no significant irregular lumping of births around the date of August 31. More formally, we implemented a test suggested by Calonico, Cattaneo, and Titiunik^19^ on the density of birth dates and found no evidence for parent manipulation.

**Figure 2.**
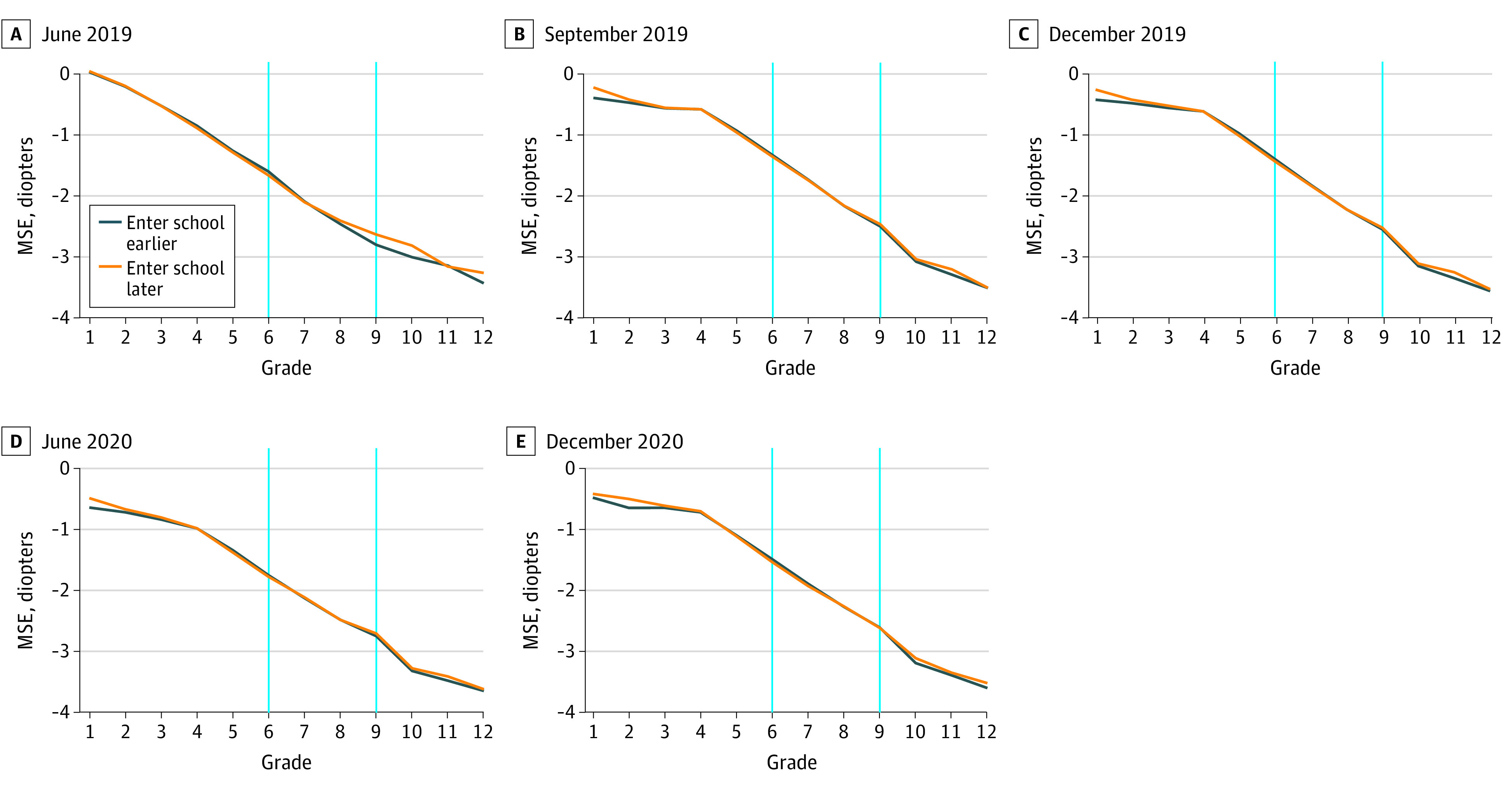
Decrease in Mean Spherical Equivalent Refractive Error (MSE) by Timing of School Entry Blue lines indicate grades 6 and 9. Entering school earlier refers to students born in January to August. When considering at the grade level, MSE was the same, if not worse, than among individuals in the control group, even if they arrived at each grade at younger ages because they entered school 1 year earlier.

Figure 2 shows that age, the core confounding factor, was not a confounder in the association of school education with myopia. When looking at youths in the same grade (ie, with the same No. of years of education), those who entered school 1 year later (ie, with older age because they were born during September and December) had the same if not better levels of MSE.

To further assess the validity of the RD design and exclude confounding factors, we plotted student characteristics against birth dates. For environmental factors, it is acknowledged that sex, intensive near work, outdoor activities, and light intensity are factors associated with myopia.^22^ If these factors were associated with students’ birth dates, then estimates of our RD design may be biased owing to inadequate controls and unobserved missing variables. However, the results of RD estimation would be more robust if this was not the case. Owing to data limitations, we tested 2 confounding risk factors: sex and intensive near work. We used studying at an intensive school as a proxy for intensive near work. In eFigure 6 and eFigure 7 in the Supplement, these factors associated with increased risk are plotted against birth dates, with 2 local linear fits on both sides of the cutoff point. There was no statistically significant discrete increase at the cutoff point. In other words, these well-acknowledged factors associated with increased risk were not specific to birth months. Further falsification testing also suggests this (eTable 3 in the Supplement).

#### Estimation Based on RD Design

**Figure 3.**
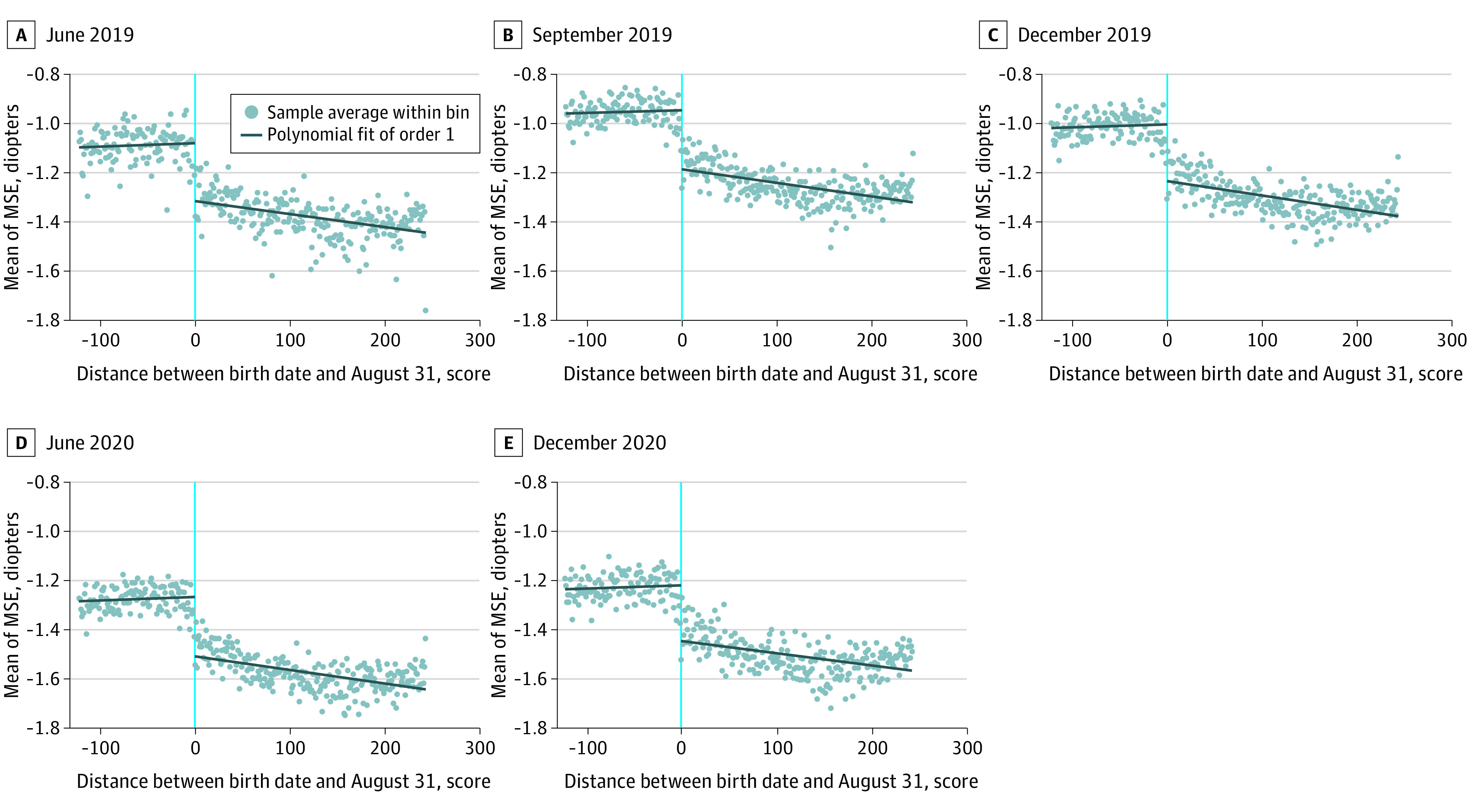
Regression Discontinuity for Mean Spherical Equivalent Refractive Error (MSE) Vertical line (zero) indicates the birth date August 31. Arithmetic means of MSE were calculated within bins by birth date. Students born after August are at the left. Polynomial linear regression was used to fit the sample separately at both sides of August 31.

Figure 3 presents a graphical analysis of our RD estimation based on MSE, with 2 local linear fit lines on both sides of the cutoff date (UVA is presented in eFigure 8 in the Supplement). On the horizontal axis, the treatment group with 1 more year of education is at the right side of the cutoff date. Therefore, if 1 year of education was associated with youth eyesight, there would be a discontinuous jump at the cutoff date, which is the case.

The first rows of Table 1 and Table 2 present RD estimates on the association of 1 year of education with youth eyesight. In odd-numbered columns, we estimated results without controlling for individual characteristics (ie, sex and studying at an intensive school) for 5 cross sections, while for even-numbered columns, we controlled these characteristics to check for robustness. All results were calculated using 35-day bandwidths (with increases and decreases of approximately 2 days for different cross sections) based on the automatic selection process suggested by Calonico, Cattaneo, and Titiunik.^19^ Standard errors were clustered at every birth date as recommend by Lee and Card to adjust for serial association and heterogeneities.^21^

**Table 1.** Association of 1 Year of Education With Mean Spherical Equivalent Refractive Error

Variable	RD estimation coefficient (*t* statistic) [95% CI], diopters^a^^,^^b^																	
	June 2019				September 2019				December 2019				June 2020				December 2020	
	Not controlled		Controlled		Not controlled		Controlled		Not controlled		Controlled		Not controlled		Controlled		Not controlled	Controlled
Conventional	−0.209 (−5.939) [−0.278 to −0.140]	−0.207 (−5.858) [−0.276 to −0.138]		−0.163 (−6.281) [−0.214 to −0.112]		−0.162 (−6.238) [−0.213 to −0.111]		−0.149 (−5.020) [−0.207 to −0.0909]		−0.148 (−4.983) [−0.206 to −0.0896]		−0.154 (−5.851) [−0.205 to −0.102]		−0.152 (−5.805) [−0.203 to −0.101]		−0.162 (−6.141) [−0.213 to −0.110]		−0.159 (−6.084) [−0.211 to −0.108]
Bias corrected	−0.197 (−5.617) [−0.266 to −0.129]	−0.196 (−5.531) [−0.265 to −0.126]		−0.158 (−6.089) [−0.209 to −0.107]		−0.157 (−6.045) [−0.208 to −0.106]		−0.143 (−4.807) [−0.201 to −0.0846]		−0.141 (−4.768) [−0.199 to −0.0832]		−0.146 (−5.569) [−0.198 to −0.0947]		−0.144 (−5.519) [−0.195 to −0.0930]		−0.155 (−5.893) [−0.207 to −0.103]		−0.153 (−5.836) [−0.204 to −0.102]
Robust	−0.197 (−4.856) [−0.277 to −0.118]	−0.196 (−4.777) [−0.276 to −0.115]		−0.158 (−5.207) [−0.217 to −0.0985]		−0.157 (−5.171) [−0.216 to −0.0974]		−0.143 (−4.092) [−0.211 to −0.0744]		−0.141 (−4.060) [−0.209 to −0.0731]		−0.146 (−4.807) [−0.206 to −0.0866]		−0.144 (−4.769) [−0.204 to −0.0850]		−0.155 (−5.080) [−0.215 to −0.0952]		−0.153 (−5.027) [−0.212 to −0.0933]
Observations	740 201	740 201		820 869		820 869		796 848		796 848		810 935		810 935		896 044		896 044

Abbreviation: RD, regression discontinuity.

^a^ Statistical significance was calculated based on *t* test. All *P* values < .001.

^b^ Column titles represent census period and control variables. For example, the column June 2019 indicates that the analysis is based on eyesight census in June 2019. Not controlled indicates that control variables, including sex, birth year, and intensive school, were not added, while controlled indicates that control variables were added. Birth year is controlled for each regression.

**Table 2.** Association of 1 Year of Education With Uncorrected Visual Acuity

Variables	RD estimation coefficient (*t* statistic) [95% CI]^a^^,^^b^									
	June 2019		September 2019		December 2019		June 2020		December 2020	
	Not controlled	Controlled	Not controlled	Controlled	Not controlled	Controlled	Not controlled	Controlled	Not controlled	Controlled
Conventional	−0.0338 (−5.757) [−0.0453 to −0.0223]	−0.0331 (−5.544) [−0.0447 to −0.0214]	−0.0274 (−5.977) [−0.0364 to −0.0184]	−0.0270 (−5.828) [−0.0361 to −0.0179]	−0.0294 (−5.666) [−0.0395 to −0.0192]	−0.0288 (−5.561) [−0.0389 to −0.0186]	−0.0307 (−6.144) [−0.0404 to −0.0209]	−0.0300 (−5.986) [−0.0398 to −0.0201]	−0.0283 (−7.189) [−0.0360 to −0.0206]	−0.0275 (−6.899) [−0.0353 to −0.0197]
Bias corrected	−0.0323 (−5.498) [−0.0438 to −0.0208]	−0.0315 (−5.291) [−0.0432 to −0.0199]	−0.0263 (−5.720) [−0.0353 to −0.0173]	−0.0258 (−5.579) [−0.0349 to −0.0168]	−0.0289 (−5.572) [−0.0391 to −0.0187]	−0.0283 (−5.465) [−0.0384 to −0.0181]	−0.0294 (−5.883) [−0.0391 to −0.0196]	−0.0287 (−5.726) [−0.0385 to −0.0188]	−0.0273 (−6.938) [−0.0350 to −0.0196]	−0.0266 (−6.656) [−0.0344 to −0.0187]
Robust	−0.0323 (−4.751) [−0.0456 to −0.0190]	−0.0315 (−4.570) [−0.0451 to −0.0180]	−0.0263 (−4.900) [−0.0368 to −0.0158]	−0.0258 (−4.777) [−0.0365 to −0.0152]	−0.0289 (−4.701) [−0.0409 to −0.0168]	−0.0283 (−4.612) [−0.0403 to −0.0163]	−0.0294 (−5.109) [−0.0406 to −0.0181]	−0.0287 (−4.970) [−0.0400 to −0.0174]	−0.0273 (−5.920) [−0.0364 to −0.0183]	−0.0266 (−5.665) [−0.0357 to −0.0174]
Observations	740 201	740 201	820 869	820 869	796 848	796 848	810 935	810 935	896 044	896 044

Abbreviation: RD, regression discontinuity.

^a^ Statistical significance is calculated based on *t* test. All *P* values < .001.

^b^ Column titles represent census period and control variables. For example, the column June 2019 indicates that the analysis is based on eyesight census in June 2019. Not controlled indicates that control variables, including sex, birth year, and intensive school, were not added, while controlled indicates that control variables were added. Birth year is controlled for each regression.

Education was associated with eyesight. We found that 1 year of education was associated with a decrease in MSE of −0.17 diopters/y (95% CI, −0.22 to −0.11 diopters/y), ranging from −0.148 diopters (95% CI, −0.206 to −0.0896 diopters) for the controlled December 2019 census to −0.209 diopters (95% CI, −0.278 to −0.140 diopters) for the uncontrolled June 2019 census (Table 1). Each year of education was associated with a decrease in UVA of −0.03 points/y (95% CI, −0.04 to −0.02 points/y), ranging from −0.027 points (95% CI, −0.036 to −0.018 points) for the controlled September 2019 census to −0.034 points (95% CI, −0.045 to −0.022 points) for the uncontrolled June 2019 census (Table 2). After receiving 12 years of education at school, student eyesight would decrease by 2.04 diopters and 0.36 points for MSE and UVA, respectively. Controlling other individual specific characteristics did not change the results.

Another 2 robustness checks were performed by adding a bias term (the second row in Table 1 and Table 2) and further adjusting variability of the bias-corrected point estimator (the third row in Table 1 and Table 2). These estimations improve the boundary properties of the conventional estimation for more robust results. The coefficients and 95% CIs changed when using different estimation methods. For example, for the June 2019 census with no controlling for individual characteristics, the RD was −0.209 diopters (95% CI, −0.278 to −0.140 diopters) in conventional analysis, −0.197 diopters (95% CI, −0.266 to −0.129 diopters) in bias-corrected analysis, and −0.197 diopters (95% CI, −0.277 to −0.118 diopters) in robust analysis (Table 1). Estimations based on pooled census data had similar results (eTable 4 in the Supplement). These results suggest that RD estimates were stable.

eTable 5 in the Supplement shows mean MSE and UVA by grade and census. The δ calculator reports shifts by grade, representing the cumulative association of all factors with eyesight among students in a grade year. The mean (SD) decrease over all grade levels was −0.256 (0.226) diopters for MSE and −0.038 (0.041) points for UVA. This suggests that the association of school education with eyesight may account for 0.17 of 0.256 diopters/y (66.4% [95% CI, 85.9% to 43.0%]) for MSE change and 0.03 of 0.038 points/y (78.9% [95% CI, 102.6% to 55.3%]) for UVA change among all factors in a grade year (eTable 6 in the Supplement).

To investigate the dynamic shift in MSE across grades and provide more insights for intervention polices, a decomposing estimator is given in eFigure 9 in the Supplement. Generally, the levels of risk factors associated with myopia increased with levels of education; however, the greatest MSE shifts occurred in grades 3 and 7 (eg, for the June 2019 census, the RD was −0.26 diopters/y [95% CI, −0.33 to −0.20 diopters/y] for grade 3 and −0.41 diopters/y [95% CI, −0.66 to −0.15 diopters/y] for grade 7). However, primary school grades experienced higher mean decreases in MSE and therefore had larger potentials for further interventions.

#### Factors Associated With School Education and Myopia

Other factors in the association between school education and myopia were not exhaustively investigated in our study owing to data limitations. However, it can be shown through eFigure 3 in the Supplement that education practices, such as preparing for examinations, may be important because the eyesight of youths was worst in June (when the final school examination occurs) and best in September (when there is no school examination).

Moreover, pressure of education and competition may be another factor. The prevalence and severity of myopia is worse in East Asia and Southeast Asia than in European areas, which coincides with the occurrence of higher-pressure education and competition.^22^ Additional evidence might be provided through Chinese zodiac preference, which poses higher competition for youth with preferred zodiac signs given that more births occur in years with those signs. The results estimated by zodiac years are plotted in eFigure 10 and summarized in eTable 7 in the Supplement.

After the outbreak of COVID-19, all schools were shut down in China, requiring students to have classes online until late April in 2020.^23^ By comparing RD estimation coefficient of MSE from the uncontrolled census of December 2020 (−0.162 diopters [95% CI, −0.213 to −0.110 diopters]) to that of 2019 (−0.149 diopters [95% CI, −0.207 to −0.091]), our RD estimation found a −0.013 (8.7%) decrease. Differences in RD estimations between June 2020 and June 2019 were not used here because students who entered school later experienced the same duration of online classes compared with students who entered school earlier.

## Discussion

Myopia is becoming increasingly prevalent worldwide, with lengthening course of disease and increased risks of blindness, associated with deficiencies in precision instrument operation and inconveniences in daily life, such as driving without glasses.^15,22,24^ Moreover, the potential association of education with eyesight also plays an important role in many economic models on human capital,^25^ because more years spent studying with intense education modes may be associated with depreciated human health capital. Agreeing with substantial existing literature,^15,26,27^ our cross-sectional study found that myopia was more severe in youths who received more years of education. The RD design was associated with reduced confounding, with factors randomly assigned to both sides of the cutoff point of the birth date August 31. If confounding factors, including sex, near work, time spent outdoors, socioeconomic position, and genetic basis, are not associated with birth dates, this suggests that the RD design may be well-grounded, without posing unethical assignments on youth education. Mountjoy et al^15^ found that every additional year of education was associated with a decrease in MSE of −0.27 diopters/y. Their study overestimated the association because the study design, mendelian randomization, belongs to instrumental studies with unavoidable bias. The estimation given by Mountjoy et al^15^ would suggest that 12 years of schooling would be associated with a shift in MSE of −3.24 diopters, suggesting that there should be higher prevalence of myopia in the United Kingdom than in China, which is not the case.

We found that education was associated with a greater decrease in MSE and UVA.^15^ Potential mediators in the association between school education and myopia are supported by our study, including preparing for examinations, pressure and competition, and longer digital screen time. After COVID-19, the decrease in MSE associated with1 year school decreased from −0.149 in the uncontrolled December 2019 census to 0.162 in the uncontrolled December 2020 census. The decrease may be associated with students using digital screens for a longer time during COVID-19 because they took classes online. Longer digital screen time seems to parallel more myopic refractive powers and may be another mediator in the association between school education and myopia instead of having uncertain associations.^28^ However, the scale of the outcome associated with digital screen time may be underestimated because the pressure of education and examination were alleviated simultaneously out of administrative arrangements and inadequate preparation during the pandemic.^29^ This suggests that our RD estimation may be a higher bound for the association of each additional year of school education with youth eyesight worldwide because pressure and competition owing to school exams are among the highest in East Asia, including China.

### Implications for Clinicians and Policy Makers

Our results suggest that intervention in the early years may be important to control myopia progression. Students in primary school grades experienced a higher mean decrease in eyesight, especially those in grade 3.

### Limitations

This study has several limitations. These include not using cycloplegic refraction when measuring MSE, which may suggest that we overestimated the prevalence of myopia. However, this may not pose serious measurement errors for this study because it was the annual shift that was of interest instead of absolute values. Additionally, thorough analysis on mediators in the association between school education and eyesight was limited owing to the lack of data.

## Conclusions

This study found that receiving more years of school education, not age, was associated with myopia among youths. Our findings suggest that examinations, education pressures, and use of digital screens may play a role in the association.

As in East Asia and Southeast Asia, North America and Europe present an upward trend of myopia. Its burden of disease, as well as its potential blinding pathologies, suggest that more research needs to be done to investigate mediators in the association between school education and myopia. For policy makers, changes in the current education mode by reducing study burden in early years of learning may alleviate the status quo of myopia management and bring improved education outcomes.

## Notes

Supplement. **eTable 1.** Process of Sample Restriction **eTable 2.** Summary Statistics **eTable 3.** Falsification Test on Covariate Intensive School **eTable 4.** Regression Discontinuity Pooled Regression **eTable 5.** Mean of Uncorrected Visual Acuity and Mean Spherical Equivalent Refractive Error by Grade **eTable 6.** Mean of Uncorrected Visual Acuity and Mean Spherical Equivalent Refractive Error by Birth Year **eTable 7.** Regression Discontinuity Regression by Chinese Zodiac (Birth Year) **eMethods.** Analytical Solution of Regression Discontinuity Estimation **eFigure 1.** Youths in Census of Myopia in Wenzhou, China, Passing Validation of Regression Discontinuity Design **eFigure 2.** Mean Uncorrected Visual Acuity of 800 000 Participants by Grade for Each Census **eFigure 3.** Mean of Mean Spherical Equivalent Refractive Error of 800 000 Participants by Birth Month for Each Census **eFigure 4.** Mean Uncorrected Visual Acuity of 800 000 Participants by Birth Month for Each Census **eFigure 5.** Frequencies of Birth by Birth Date for Each Census **eFigure 6.** Regression Discontinuity Plot by Sex **eFigure 7.** Regression Discontinuity Plot by Admittance to Intensive School **eFigure 8.** Regression Discontinuity Plot for Uncorrected Visual Acuity **eFigure 9.** Regression Discontinuity Coefficients by Grade **eFigure 10.** Coefficients by Chinese Zodiac Year (Birth Year)
eReferences.
