# Errors in Author Degrees and Affiliations

**DOI:** 10.1001/jamanetworkopen.2022.19315

**Published:** 2022-06-13

In the Original Investigation titled “Association of School Education With Eyesight Among Children and Adolescents,”^1^ published April 29, 2022, there were errors in the degrees and affiliations for 1 author. This article has been corrected.^1^
