# Supplementary material for: Association of School Education With Eyesight Among Children and Adolescents
Source: JAMA Netw Open. 2022 Apr 29;5(4):e229545. doi: 10.1001/jamanetworkopen.2022.9545 (PMC9055461; doi:10.1001/jamanetworkopen.2022.9545)
Supplement: Supplement. — eTable 1. Process of Sample Restriction eTable 2. Summary Statistics eTable 3. Falsification Test on Covariate Intensive School eTable 4. Regression Discontinuity Pooled Regression eTable 5. Mean of Uncorrected Visual Acuity and Mean Spherical Equivalent Refractive Error by Grade eTable 6. Mean of Uncorrected Visual Acuity and Mean Spherical Equivalent Refractive Error by Birth Year eTable 7. Regression Discontinuity Regression by Chinese Zodiac (Birth Year) eMethods. Analytical Solution of Regression Discontinuity Estimation eFigure 1. Youths in Census of Myopia in Wenzhou, China, Passing Validation of Regression Discontinuity Design eFigure 2. Mean Uncorrected Visual Acuity of 800 000 Participants by Grade for Each Census eFigure 3. Mean of Mean Spherical Equivalent Refractive Error of 800 000 Participants by Birth Month for Each Census eFigure 4. Mean Uncorrected Visual Acuity of 800 000 Participants by Birth Month for Each Census eFigure 5. Frequencies of Birth by Birth Date for Each Census eFigure 6. Regression Discontinuity Plot by Sex eFigure 7. Regression Discontinuity Plot by Admittance to Intensive School eFigure 8. Regression Discontinuity Plot for Uncorrected Visual Acuity eFigure 9. Regression Discontinuity Coefficients by Grade eFigure 10. Coefficients by Chinese Zodiac Year (Birth Year) eReferences. [file jamanetwopen-e229545-s001.pdf]

Supplemental Online Content

Zhang C, Li L, Jan C, Qu J. The association of school education with eyesight among children and adolescents. *JAMA Netw Open.* 2022;5(4):e229545.
doi:10.1001/jamanetworkopen.2022.9545

eTable 1. Process of Sample Restriction
eTable 2. Summary Statistics
eTable 3. Falsification Test on Covariate Intensive School
eTable 4. Regression Discontinuity Pooled Regression
eTable 5. Mean of Uncorrected Visual Acuity and Mean Spherical Equivalent Refractive Error by Grade
eTable 6. Mean of Uncorrected Visual Acuity and Mean Spherical Equivalent Refractive Error by Birth Year
eTable 7. Regression Discontinuity Regression by Chinese Zodiac (Birth Year)
eMethods. Analytical Solution of Regression Discontinuity Estimation
eFigure 1. Youths in Census of Myopia in Wenzhou, China, Passing Validation of Regression Discontinuity Design
eFigure 2. Mean Uncorrected Visual Acuity of 800 000 Participants by Grade for Each Census
eFigure 3. Mean of Mean Spherical Equivalent Refractive Error of 800 000 Participants by Birth Month for Each Census
eFigure 4. Mean Uncorrected Visual Acuity of 800 000 Participants by Birth Month for Each Census
eFigure 5. Frequencies of Birth by Birth Date for Each Census
eFigure 6. Regression Discontinuity Plot by Sex
eFigure 7. Regression Discontinuity Plot by Admittance to Intensive School
eFigure 8. Regression Discontinuity Plot for Uncorrected Visual Acuity
eFigure 9. Regression Discontinuity Coefficients by Grade
eFigure 10. Coefficients by Chinese Zodiac Year (Birth Year)
eReferences.

This supplemental material has been provided by the authors to give readers additional information about their work.

© 2022 Zhang C et al. *JAMA Network Open.*

eTable 1. Process of Sample Restriction

	201906	201909	201912	202006	202012
Whole Sample	1002626	1010913	979214	996127	1024649
Restrict Age (6-18) Sample	1002068	1009846	978239	995216	1023655
Restrict Comparability Sample	934827	948799	919756	934828	962805
RD Sample	740201	820869	796848	810935	896044

Note: The number of participants are reported. Column titles represent census period. For example, the column of 201906 means the analysis is based on eyesight census at June 2019. Whole Sample is the raw data. Restrict Age (6-18) Sample deletes students whose age is beyond 6-18 because this limited number of observations bring a significant bias to the descriptive analysis. Restrict Comparability Sample deletes students who were born before September 1st and just entered the first grade at that year because they do not have comparable students born in or after September included in the sample. And we do the same for those born after August 31st just began their last year of education at senior high school. The Restrict Comparability Sample is used for summary statistics and related figures. The RD Sample further deletes students born before and in the year of 2003 because of the change of implementation of China's Compulsory Education Law. This sample is used for regression discontinuity analysis.

© 2022 Zhang C et al. *JAMA Network Open.*

eTable 2. Summary Statistics

	201906						201909						201912				
	(1)	(2)	(3)	(4)	(5)		(1)	(2)	(3)	(4)	(5)		(1)	(2)	(3)	(4)	(5)
VARIABLES	N	mean	sd	min	max		N	mean	sd	min	max		N	mean	sd	min	max
Grade	934827	6.188	3.135	1.000	12.000		948799	5.584	3.074	1.000	12.000		919756	5.577	3.067	1.000	12.000
Female	934827	0.450	0.498	0.000	1.000		948799	0.451	0.498	0.000	1.000		919756	0.451	0.498	0.000	1.000
Birth Year	934827	-	-	2,000	2,011		948799	-	-	2,001	2,012		919756	-	-	2,001	2,012
Intensive	934827	0.204	0.403	0.000	1.000		948799	0.203	0.402	0.000	1.000		919756	0.201	0.401	0.000	1.000
Uncorrected Visual Acuity	934827	4.711	0.362	0.000	5.300		948799	4.756	0.348	0.800	5.300		919756	4.755	0.349	3.300	5.300
Spherical Power	934827	-1.320	7.289	-3,536.125	1,013.375		948799	-1.050	2.259	-1,013.625	103.500		919756	-1.117	1.985	-18.875	16.000
Cylindrical Power	934827	-0.674	2.213	-1,013.000	0.000		948799	-0.694	0.758	-91.125	0.000		919756	-0.662	0.654	-8.625	0.000
Axial	934827	91.386	86.333	-180.000	66,360.000		948799	90.586	51.456	-86.500	591.000		919756	88.859	50.636	0.000	180.000

© 2022 Zhang C et al. *JAMA Network Open.*

MSE	934827	-1.657	7.252	-3,537.000	506.875		948799	-1.396	2.301	-1,013.625	103.375		919756	-1.448	2.063	-19.500	15.312

© 2022 Zhang C et al. *JAMA Network Open.*

eTable 2. Summary Statistics (continued)

	202006						202012				
	(1)	(2)	(3)	(4)	(5)		(1)	(2)	(3)	(4)	(5)
VARIABLES	N	mean	sd	min	max		N	mean	sd	min	max
Grade	934828	5.596	3.082	1.000	12.000		962805	5.639	3.071	1.000	12.000
Female	934828	0.451	0.498	0.000	1.000		962805	0.451	0.498	0.000	1.000
Birth Year	934828	-	-	2,001	2,012		962805	-	-	2,002	2,013
Intensive	934828	0.212	0.409	0.000	1.000		962805	0.208	0.406	0.000	1.000
Uncorrected Visual Acuity	934828	4.712	0.359	3.300	5.300		962805	4.731	0.358	3.300	5.300
Spherical Power	934828	-1.334	2.002	-19.625	15.625		962805	-1.162	1.978	-18.750	16.875
Cylindrical Power	934828	-0.734	0.677	-8.625	0.000		962805	-0.744	0.678	-8.375	0.000
Axial	934828	92.440	51.886	0.000	180.000		962805	93.569	51.896	0.000	180.000
MSE	934828	-1.701	2.080	-20.438	14.000		962805	-1.534	2.056	-19.812	15.250

© 2022 Zhang C et al. *JAMA Network Open.*

**eTable 3.** Falsification Test on Covariate Intensive School					
	(1)	(2)	(3)	(4)	(5)
VARIABLES	201906	201909	201912	202006	202012
Conventional	-0.00794*	-0.00392	-0.00281	-0.00302	0.00485
	(-1.934)	(-1.270)	(-0.791)	(-0.831)	(1.356)
	[-0.0160 - 0.000108]	[-0.00998 - 0.00213]	[-0.00979 - 0.00416]	[-0.0102 - 0.00410]	[-0.00216 - 0.0119]
Bias-corrected	-0.00622	-0.00389	-0.00244	-0.00302	0.00503
	(-1.515)	(-1.257)	(-0.685)	(-0.829)	(1.406)
	[-0.0143 - 0.00183]	[-0.00994 - 0.00217]	[-0.00941 - 0.00454]	[-0.0101 - 0.00411]	[-0.00198 - 0.0120]
Robust	-0.00622	-0.00389	-0.00244	-0.00302	0.00503
	(-1.368)	(-1.057)	(-0.563)	(-0.692)	(1.174)
	[-0.0151 - 0.00270]	[-0.0111 - 0.00332]	[-0.0109 - 0.00604]	[-0.0116 - 0.00552]	[-0.00337 - 0.0134]
Observations	740,201	820,869	796,848	810,935	896,044
Birth Year	YES	YES	YES	YES	YES
Control	YES	YES	YES	YES	YES
Note: T-statistics in parentheses. 95% confidence intervals in square brackets. Statistical significance is calculated based on t-test, and then reported as *** p<0.01, ** p<0.05, * p<0.1. Column titles represent census period. For example, the column of 201906 means the analysis is based on eyesight census at June 2019.					

© 2022 Zhang C et al. *JAMA Network Open.*

**eTable 4.** Regression Discontinuity Pooled Regression				
	(1)	(2)	(3)	(4)
VARIABLES	UVA	UVA Cov	MSE	MSE Cov
Conventional	-0.0299***	-0.0293***	-0.167***	-0.165***
	(-6.375)	(-6.188)	(-6.114)	(-6.067)
	[-0.0391 - -0.0207]	[-0.0385 - -0.0200]	[-0.220 - -0.113]	[-0.219 - -0.112]
Bias-corrected	-0.0288***	-0.0282***	-0.160***	-0.158***
	(-6.137)	(-5.953)	(-5.867)	(-5.817)
	[-0.0380 - -0.0196]	[-0.0374 - -0.0189]	[-0.214 - -0.107]	[-0.212 - -0.105]
Robust	-0.0288***	-0.0282***	-0.160***	-0.158***
	(-5.284)	(-5.124)	(-5.040)	(-4.997)
	[-0.0395 - -0.0181]	[-0.0389 - -0.0174]	[-0.222 - -0.0979]	[-0.220 - -0.0963]
Observations	4,064,897	4,064,897	4,064,897	4,064,897
Birth Year	YES	YES	YES	YES
Census	YES	YES	YES	YES
Control	NO	YES	NO	YES

Note: T-statistics in parentheses. 95% confidence intervals in square brackets. Statistical significance is calculated based on t-test, and then reported as *** p<0.01, ** p<0.05, * p<0.1. Data from different census periods are pooled here. Column titles represent the measure of eyesight and control variables. For example, the column of UVA means the analysis is based on uncorrected visual acuity, without adding control variables including gender, birth year, and intensive school, while the column of UVA Cov means the analysis is based on uncorrected visual acuity, with adding control variables including gender, birth year, and intensive school.

© 2022 Zhang C et al. *JAMA Network Open.*

**eTable 5.** Mean of Uncorrected Visual Acuity and Mean Spherical Equivalent Refractive Error by Grade												
	(1)	(2)	(3)	(4)	(5)	(6)	(7)	(8)	(9)	(10)	(11)	(12)
	grade 1	grade 2	grade 3	grade 4	grade 5	grade 6	grade 7	grade 8	grade 9	grade 10	grade 11	grade 12
VARIABLES	mean	mean	mean	mean	mean	mean	mean	mean	mean	mean	mean	mean
Panel A: 201906												
Vision	4.964	4.934	4.894	4.838	4.772	4.716	4.633	4.587	4.554	4.507	4.486	4.475
MSE	0.057	-0.179	-0.501	-0.844	-1.254	-1.610	-2.087	-2.436	-2.743	-2.938	-3.148	-3.379
Delta of Vision	-	-0.03	-0.04	-0.056	-0.066	-0.056	-0.083	-0.046	-0.033	-0.047	-0.021	-0.011
Delta of MSE	-	-0.236	-0.322	-0.343	-0.41	-0.356	-0.477	-0.349	-0.307	-0.195	-0.21	-0.231
Panel B: 201909												
Vision	4.826	4.909	4.903	4.896	4.841	4.773	4.701	4.639	4.588	4.472	4.453	4.420
MSE	-0.783	-0.424	-0.523	-0.551	-0.917	-1.321	-1.724	-2.151	-2.475	-3.056	-3.254	-3.506
Delta of Vision	-	0.083	-0.006	-0.007	-0.055	-0.068	-0.072	-0.062	-0.051	-0.116	-0.019	-0.033
Delta of MSE	-	0.359	-0.099	-0.028	-0.366	-0.404	-0.403	-0.427	-0.324	-0.581	-0.198	-0.252
Panel B: 201912												
Vision	4.844	4.915	4.904	4.893	4.834	4.767	4.695	4.630	4.578	4.474	4.456	4.434
MSE	-0.833	-0.444	-0.522	-0.601	-0.980	-1.404	-1.821	-2.222	-2.544	-3.144	-3.324	-3.563

© 2022 Zhang C et al. *JAMA Network Open.*

Delta of Vision	-	0.071	-0.011	-0.011	-0.059	-0.067	-0.072	-0.065	-0.052	-0.104	-0.018	-0.022
Delta of MSE	-	0.389	-0.078	-0.079	-0.379	-0.424	-0.417	-0.401	-0.322	-0.6	-0.18	-0.239
Panel B: 202006												
Vision	4.835	4.884	4.860	4.827	4.770	4.703	4.638	4.582	4.547	4.453	4.438	4.446
MSE	-0.998	-0.664	-0.785	-0.948	-1.323	-1.734	-2.098	-2.459	-2.714	-3.290	-3.442	-3.628
Delta of Vision	-	0.049	-0.024	-0.033	-0.057	-0.067	-0.065	-0.056	-0.035	-0.094	-0.015	0.008
Delta of MSE	-	0.334	-0.121	-0.163	-0.375	-0.411	-0.364	-0.361	-0.255	-0.576	-0.152	-0.186
Panel B: 202012												
Vision	4.813	4.891	4.891	4.869	4.808	4.747	4.663	4.612	4.554	4.461	4.433	4.417
MSE	-0.991	-0.557	-0.595	-0.681	-1.072	-1.479	-1.881	-2.246	-2.594	-3.150	-3.366	-3.568
Delta of Vision	-	0.078	0	-0.022	-0.061	-0.061	-0.084	-0.051	-0.058	-0.093	-0.028	-0.016
Delta of MSE	-	0.434	-0.038	-0.086	-0.391	-0.407	-0.402	-0.365	-0.348	-0.556	-0.216	-0.202
Note: Vision refers to the Uncorrected Visual Acuity.												

© 2022 Zhang C et al. *JAMA Network Open.*

eTable 6. Mean of Uncorrected Visual Acuity and Mean Spherical Equivalent Refractive Error by Birth Year

	(1)	(2)	(3)	(4)	(5)	(6)	(7)	(8)	(9)	(10)	(11)	(12)
Panel A: 201906	2000	2001	2002	2003	2004	2005	2006	2007	2008	2009	2010	2011
VARIABLES	mean	mean	mean	mean	mean	mean	mean	mean	mean	mean	mean	mean
Vision	4.493	4.481	4.494	4.524	4.565	4.603	4.665	4.737	4.798	4.861	4.910	4.943
MSE	-3.293	-3.260	-3.035	-2.875	-2.648	-2.327	-1.920	-1.464	-1.101	-0.712	-0.382	-0.084
Delta of Vision	-	-0.012	0.013	0.03	0.041	0.038	0.062	0.072	0.061	0.063	0.049	0.033
Delta of MSE	-	0.033	0.225	0.16	0.227	0.321	0.407	0.456	0.363	0.389	0.33	0.298
Panel B: 201909	2001	2002	2003	2004	2005	2006	2007	2008	2009	2010	2011	2012
VARIABLES	mean	mean	mean	mean	mean	mean	mean	mean	mean	mean	mean	mean
Vision	4.484	4.491	4.520	4.553	4.606	4.661	4.729	4.800	4.864	4.915	4.956	4.968
MSE	-3.143	-3.151	-2.908	-2.664	-2.365	-1.997	-1.572	-1.163	-0.771	-0.421	-0.122	0.131
Delta of Vision	-	0.007	0.029	0.033	0.053	0.055	0.068	0.071	0.064	0.051	0.041	0.012
Delta of MSE	-	-0.008	0.243	0.244	0.299	0.368	0.425	0.409	0.392	0.35	0.299	0.253
Panel C: 201912	2001	2002	2003	2004	2005	2006	2007	2008	2009	2010	2011	2012
VARIABLES	mean	mean	mean	mean	mean	mean	mean	mean	mean	mean	mean	mean
Vision	4.491	4.499	4.528	4.556	4.597	4.653	4.723	4.793	4.857	4.913	4.958	4.979
MSE	-3.194	-3.185	-2.964	-2.734	-2.431	-2.077	-1.664	-1.239	-0.828	-0.461	-0.138	0.104
Delta of Vision	-	0.008	0.029	0.028	0.041	0.056	0.07	0.07	0.064	0.056	0.045	0.021

© 2022 Zhang C et al. *JAMA Network Open.*

Delta of MSE	-	0.009	0.221	0.23	0.303	0.354	0.413	0.425	0.411	0.367	0.323	0.242
Panel D: 202006	2001	2002	2003	2004	2005	2006	2007	2008	2009	2010	2011	2012
VARIABLES	mean	mean	mean	mean	mean	mean	mean	mean	mean	mean	mean	mean
Vision	4.488	4.492	4.510	4.530	4.559	4.603	4.663	4.729	4.793	4.852	4.909	4.946
MSE	-3.321	-3.315	-3.109	-2.903	-2.625	-2.324	-1.966	-1.575	-1.170	-0.802	-0.451	-0.166
Delta of Vision	-	0.004	0.018	0.02	0.029	0.044	0.06	0.066	0.064	0.059	0.057	0.037
Delta of MSE	-	0.006	0.206	0.206	0.278	0.301	0.358	0.391	0.405	0.368	0.351	0.285
Panel E: 202012	2002	2003	2004	2005	2006	2007	2008	2009	2010	2011	2012	2013
VARIABLES	mean	mean	mean	mean	mean	mean	mean	mean	mean	mean	mean	mean
Vision	4.487	4.481	4.506	4.533	4.576	4.631	4.696	4.771	4.833	4.890	4.936	4.959
MSE	-3.213	-3.234	-3.008	-2.777	-2.461	-2.116	-1.723	-1.315	-0.918	-0.552	-0.237	-0.004
Delta of Vision	-	-0.006	0.025	0.027	0.043	0.055	0.065	0.075	0.062	0.057	0.046	0.023
Delta of MSE	-	-0.021	0.226	0.231	0.316	0.345	0.393	0.408	0.397	0.366	0.315	0.233
Note: Vision refers to the Uncorrected Visual Acuity.												

© 2022 Zhang C et al. *JAMA Network Open.*

eTable 7. Regression Discontinuity Regression by Chinese Zodiac (Birth Year)

	(1)	(2)	(3)	(4)	(5)	(6)	(7)	(8)	(9)	(10)
VARIABLES	Monkey	Rooster	Dog	Pig	Rat	OX	Tiger	Rabbit	Dragon	Snake
Conventional	-0.0328	0.115*	-0.215**	-0.0494	-0.0915**	-0.185***	-0.294***	-0.279***	-0.209***	-0.154***
	(-0.441)	(1.893)	(-2.392)	(-0.861)	(-2.239)	(-3.331)	(-6.059)	(-7.692)	(-8.588)	(-5.541)
	[-0.179 - 0.113]	[-0.00408 - 0.234]	[-0.391 - - 0.0388]	[-0.162 - 0.0631]	[-0.172 - - 0.0114]	[-0.294 - - 0.0761]	[-0.390 - - 0.199]	[-0.351 - - 0.208]	[-0.257 - - 0.161]	[-0.208 - - 0.0994]
Bias-corrected	-0.0335	0.130**	-0.213**	-0.0183	-0.0813**	-0.183***	-0.309***	-0.286***	-0.208***	-0.145***
	(-0.450)	(2.130)	(-2.369)	(-0.319)	(-1.990)	(-3.295)	(-6.354)	(-7.871)	(-8.554)	(-5.237)
	[-0.179 - 0.112]	[0.0103 - 0.249]	[-0.389 - - 0.0367]	[-0.131 - 0.0942]	[-0.161 - - 0.00124]	[-0.292 - - 0.0741]	[-0.404 - - 0.213]	[-0.357 - - 0.215]	[-0.256 - - 0.161]	[-0.200 - - 0.0910]
Robust	-0.0335	0.130*	-0.213**	-0.0183	-0.0813*	-0.183***	-0.309***	-0.286***	-0.208***	-0.145***
	(-0.381)	(1.799)	(-1.990)	(-0.271)	(-1.709)	(-2.699)	(-5.394)	(-6.781)	(-7.135)	(-4.404)
	[-0.206 - 0.139]	[-0.0116 - 0.271]	[-0.422 - - 0.00321]	[-0.150 - 0.114]	[-0.175 - 0.0119]	[-0.316 - - 0.0501]	[-0.421 - - 0.196]	[-0.368 - - 0.203]	[-0.266 - - 0.151]	[-0.210 - - 0.0807]
Observations	71,246	75,194	83,672	88,390	91,542	92,775	92,989	98,391	107,245	94,600

Note: T-statistics in parentheses. 95% confidence intervals in square brackets. Statistical significance is calculated based on t-test, and then reported as *** p<0.01, ** p<0.05, * p<0.1. Column titles represent zodiac birthyear. The one-to-one match between zodiac year and calendar year is: Monkey-2004, Rooster-2005, Dog-2006, Pig-2007, Rat-2008, Ox-2009, Tiger-2010, Rabbit-2011, Dragon-2012, Snake-2013. Gender, intensive school, and birthyear (age) are controlled.

© 2022 Zhang C et al. *JAMA Network Open.*

© 2022 Zhang C et al. *JAMA Network Open.*

eMethods. Analytical Solution of Regression Discontinuity Estimation

The RD regression model is as follows:

$$Y_i = \beta_0 + \beta_1(X_i - C) + \delta D_i + \beta_2(X_i - C)D_i + \lambda Z_i + \varepsilon_i \quad (1)$$

where $C - h < X_i < C + h$.

The subscript *i* indicates the student. Y_i represents the dependent variables: the MSE and the UVA. X_i is the running variable: birthdate, which is narrowed to a small neighborhood (about 35-day bandwidths, represented by *h*) around C which represents the date of August 31st in each year. When a student is born earlier than C, $X_i - C$ is positive while the treatment assignment indicator D_i equals one meaning the student is assigned with one more year of formal education. Z_i is the control variables including gender, birth year, and intensive school with more intensive usage of eyes. ε_i refers to the error term. Standard errors are clustered at every running variable (birthdate) values as recommended by Lee and Card.[1]

Based on equation (1), the analytical solution of δ can be derived as follows:

$$\tau(h) = \mu_+(h) - \mu_-(h) \quad (2)$$

$$\mu_+(h) = argmin \sum_{i=1}^{n} 1.(X_i \geq C)\{Y_i - \beta_{1,+}(X_i - C) + \lambda Z_i\}\text{^}2. K_{h_n}(X_i - C) \quad (3)$$

$$\mu_-(h) = argmin \sum_{i=1}^{n} 1.(X_i < C)\{Y_i - \beta_1, -(X_i - C) + \lambda Z_i\}\text{^}2. K_{h_n}(X_i - C) \quad (4)$$

where $K_{h_n}(X_i - C)$ is the triangular kernel function used for weighting by giving more weights for participants who were born nearer to the cutoff point C.

Based on data at hands, linear regression can be done to fit model (1) and then obtains estimation of δ as well as its standard error and confidence intervals. Statistical significance can be calculated through t-test.

© 2022 Zhang C et al. *JAMA Network Open.*

eFigure 1. Youths in Census of Myopia in Wenzhou, China, Passing Validation of Regression Discontinuity Design

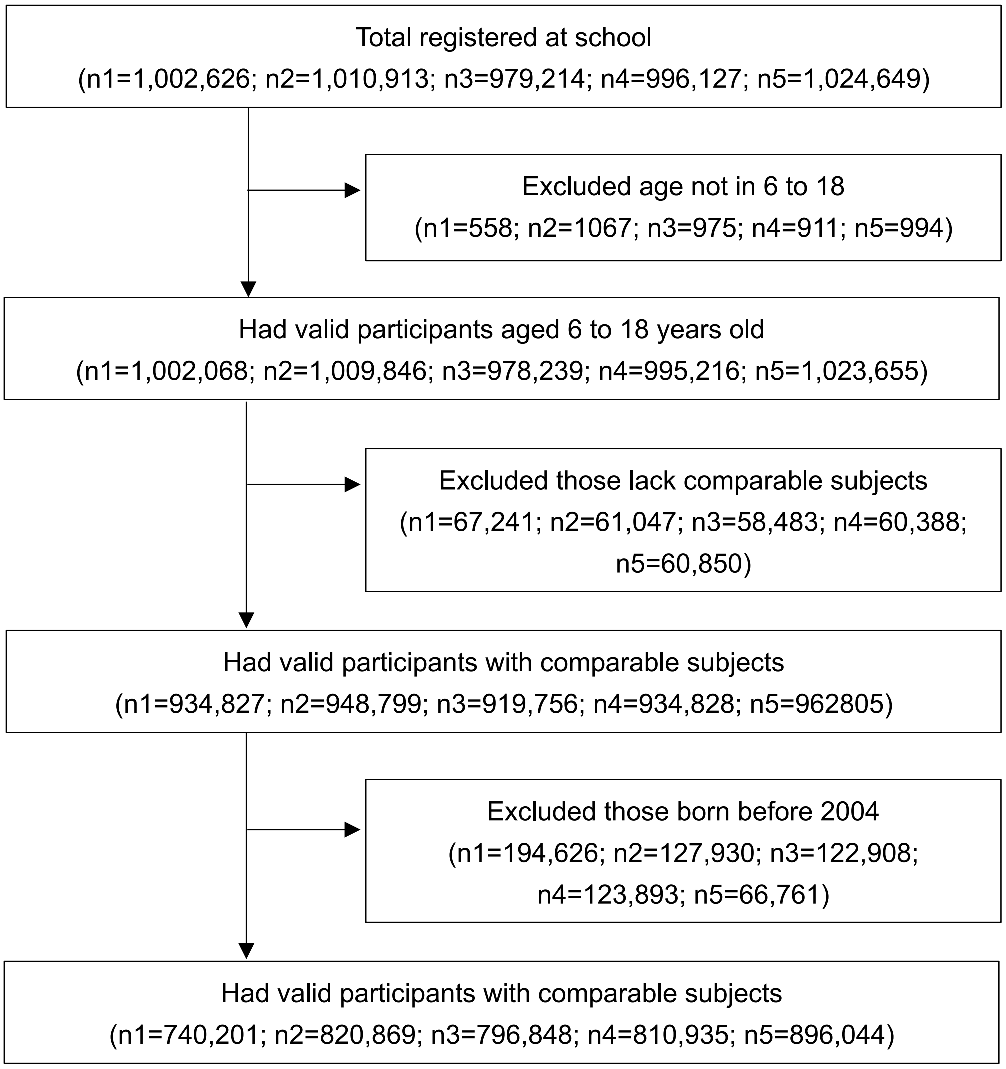

Note: n1 refers to the number of participants at the cross section of June, 2019; n2 refers to the number of participants at the cross section of September, 2019; n3 refers to the number of participants at the cross section of December, 2019; n4 refers to the number of participants at the cross section of June, 2020; n5 refers to the number of participants at the cross section of December, 2020;

© 2022 Zhang C et al. *JAMA Network Open.*

eFigure 2. Mean Uncorrected Visual Acuity of 800 000 Participants by Grade for Each Census

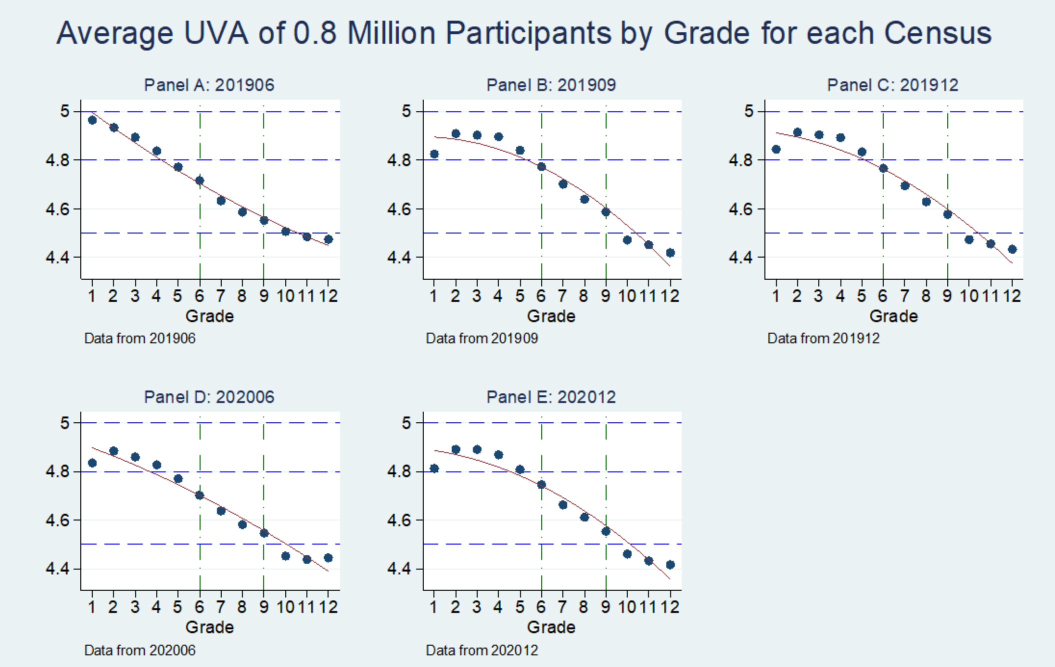

Note: UVA is uncorrected visual acuity. Arithmetic mean of UVA is calculated by grade. Panel titles represent census period. For example, the Panel A of 201906 means the calculation is based on census at June 2019. Blue lines are cutoff values of poor vision diagnosis; Green lines are Grade six and Grade nine.

© 2022 Zhang C et al. *JAMA Network Open.*

eFigure 3. Mean of Mean Spherical Equivalent Refractive Error of 800 000 Participants by Birth Month for Each Census

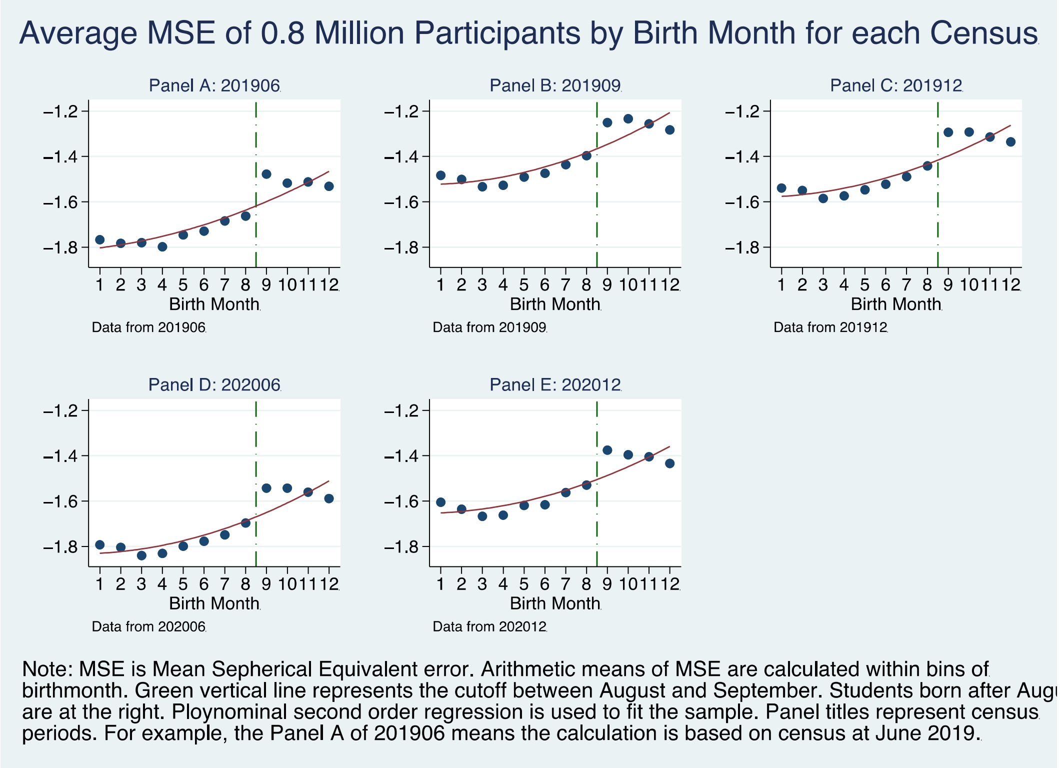

© 2022 Zhang C et al. *JAMA Network Open.*

eFigure 4. Mean Uncorrected Visual Acuity of 800 000 Participants by Birth Month for Each Census

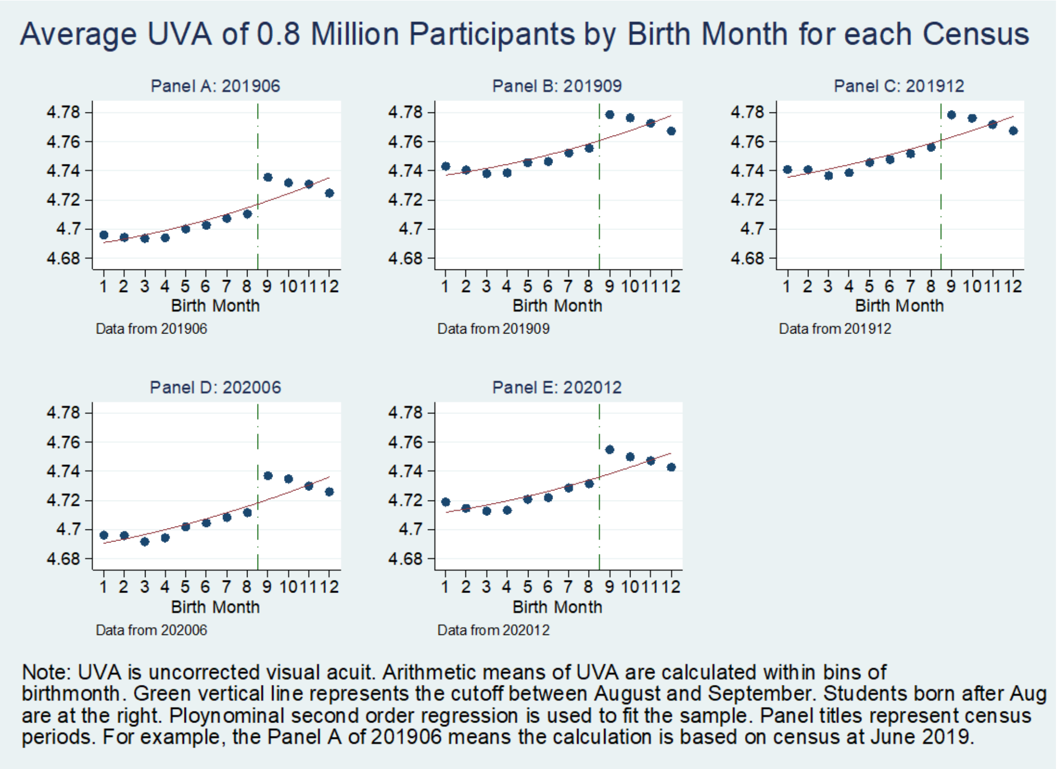

© 2022 Zhang C et al. *JAMA Network Open.*

eFigure 5. Frequencies of Birth by Birth Date for Each Census

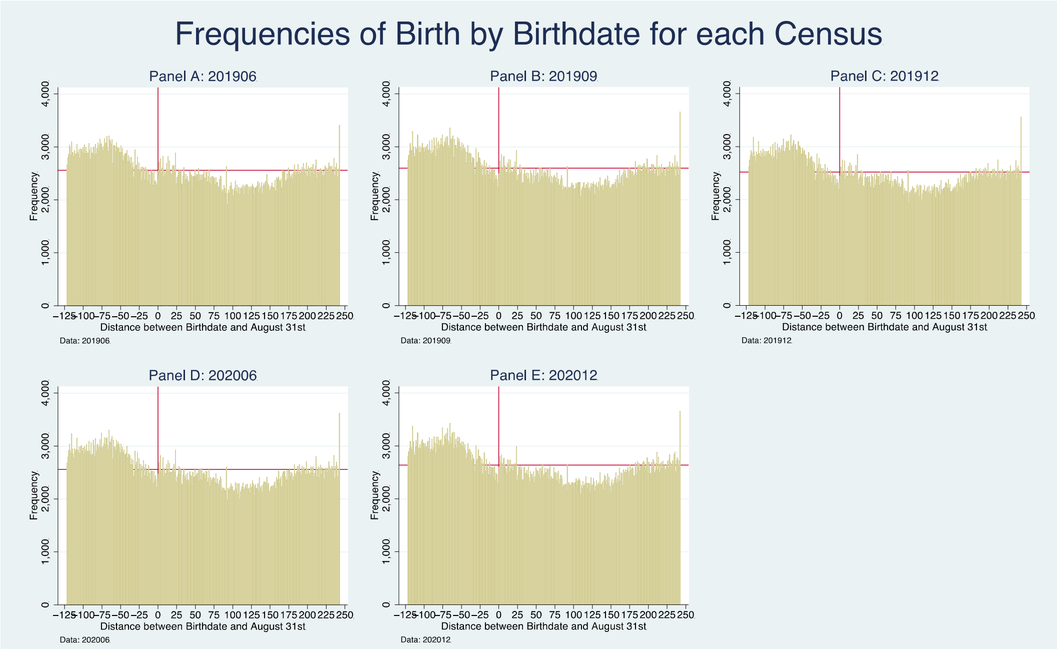

Note: Panel titles represent census periods. For example, the Panel of 201906 means the calculation is based on eyesight census at June 2019. Red vertical line (value 0 of distnace) represents the date of August 31st. Students born after August 31st are the left. Red horizontal line represents average births per date.

© 2022 Zhang C et al. *JAMA Network Open.*

eFigure 6. Regression Discontinuity Plot by Sex

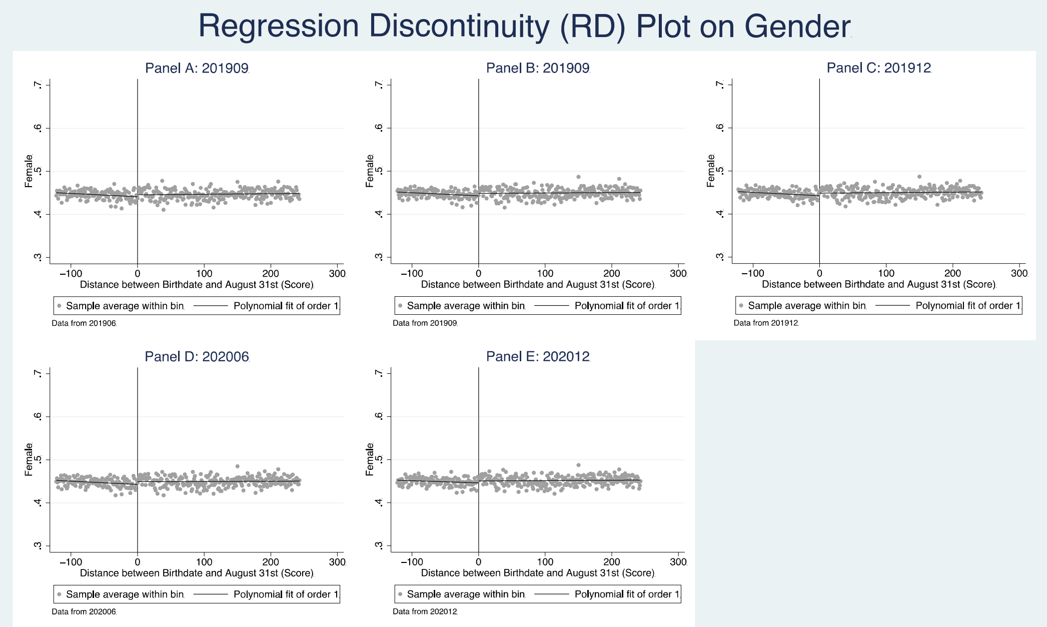

Note: Arithmetic means of Gender (female=1, male =0) are calculated within bins of birthdate. Vertical line (zero) represents the birthdate, August 31st. Students born after August are at the left. Ploynominal linear regression is used to fit the sample separately at both sides of August 31st. Panel titles represent census periods. For example, the Panel A of 201906 means the calculation is based on census at June 2019.

© 2022 Zhang C et al. *JAMA Network Open.*

eFigure 7. Regression Discontinuity Plot by Admittance to Intensive School

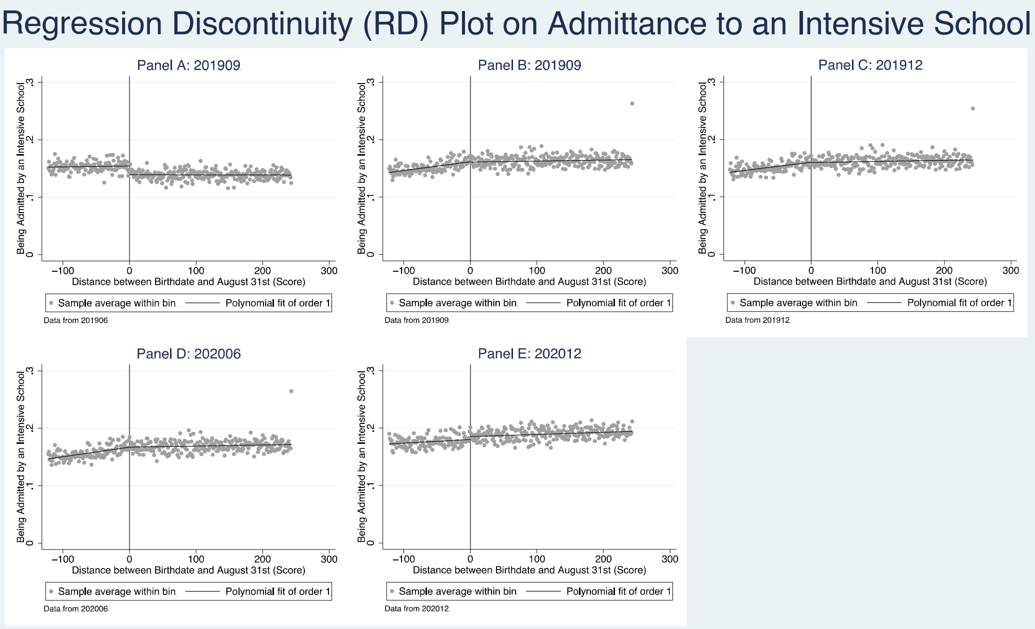

Note: Arithmetic means of being admitted to an intensive school are calculated within bins of birthdate. Vertical line (zero) represents the birthdate, August 31st. Students born after August are at the left. Ploynominal linear regression is used to fit the sample separately at both sides of August 31st. Panel titles represent census periods. For example, the Panel A of 201906 means the calculation is based on census at June 2019.

© 2022 Zhang C et al. *JAMA Network Open.*

eFigure 8. Regression Discontinuity Plot for Uncorrected Visual Acuity

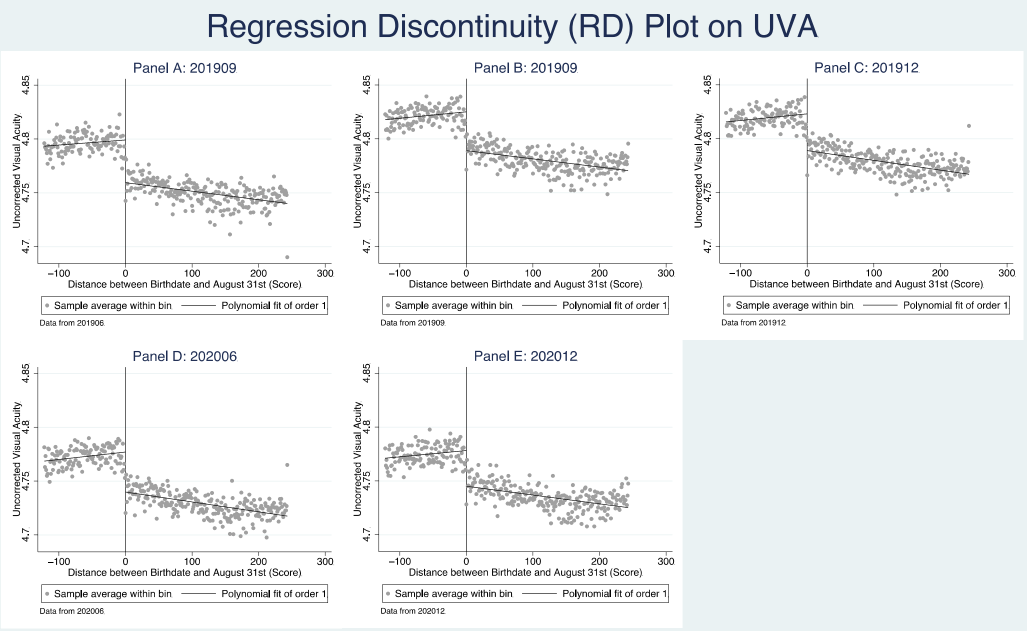

Note: UVA is uncorrected visual acuity. Arithmetic means of UVA are calculated within bins of birthdate. Vertical line (zero) represents the birthdate, August 31st. Students born after August are at the left. Ploynominal linear regression is used to fit the sample separately at both sides of August 31st. Panel titles represent census periods. For example, the Panel A of 201906 means the calculation is based on census at June 2019.

© 2022 Zhang C et al. *JAMA Network Open.*

eFigure 9. Regression Discontinuity Coefficients by Grade

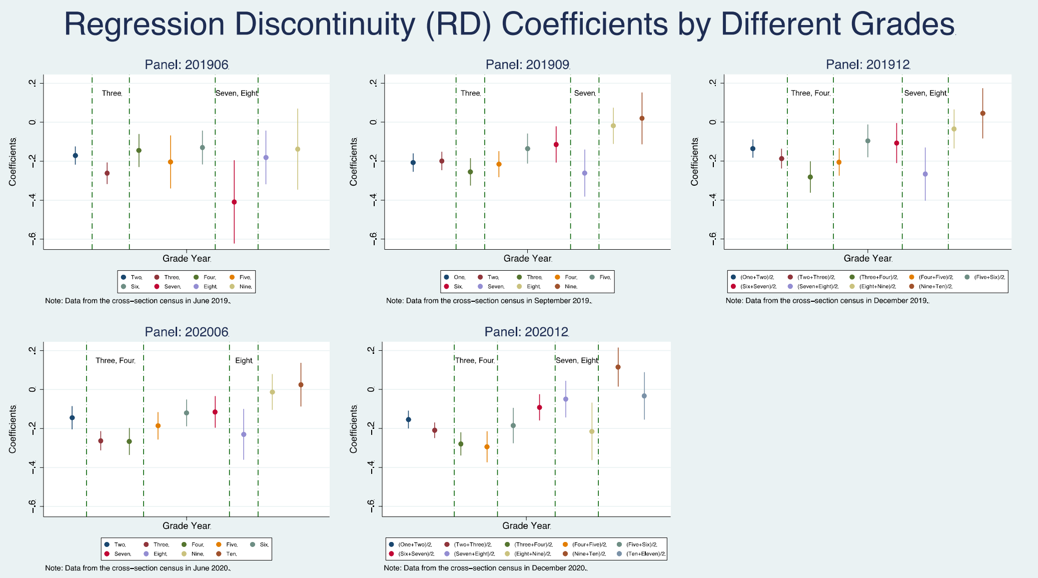

Note:T–test is used. 95% confidence intervals are reported. Panel titles represent census period. For example the Panel of 201906 means the calculation is based on eyesight census at June 2019. Each school year begins at September and ends in July the year after in China. Thus estimators from data collected at September give estimations on the association from grade one and higher; estimators from data collected at December give estimations on the association from half grade two and higher; estimators from data collected at June give estimations on the association from grade two and higher. Because of the CSL reform in 2009, the RD method can only accurately estimate the effect till Grade Eleven.

© 2022 Zhang C et al. *JAMA Network Open.*

eFigure 10. Coefficients by Chinese Zodiac Year (Birth Year)

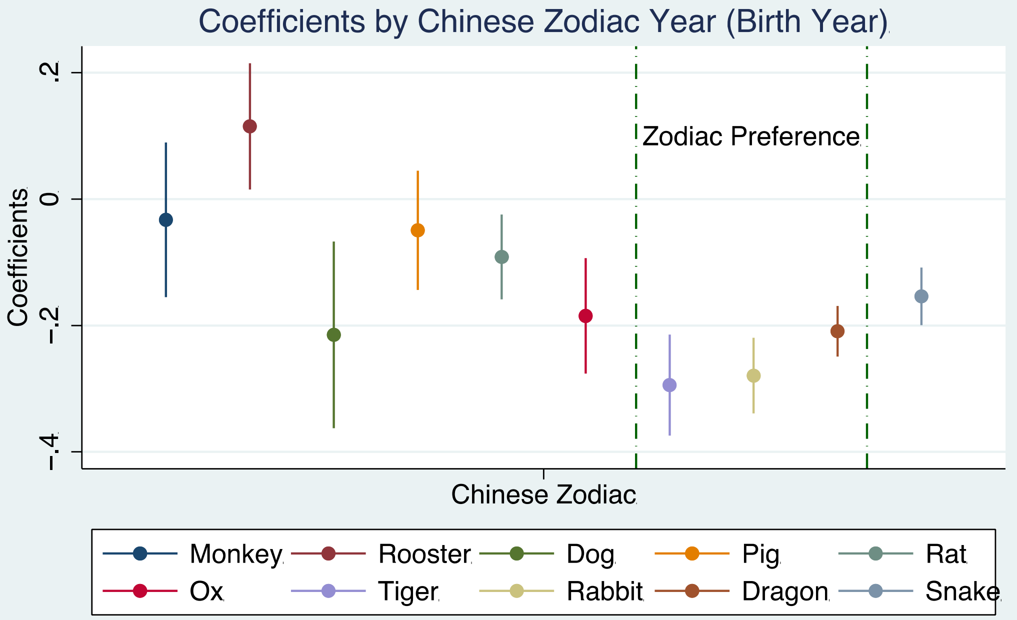

Note: T–test is used. 95% confidence intervals are reported. Data from eyesight census at December 2020 because it covers the zodiac of interest. The one–to–one match between zodiac year and calendar year is: Monkey–2004, Rooster–2005, Dog–2006, Pig–2007, Rat–2008, Ox–2009, Tiger–2010, Rabbit–2011, Dragon–2012, Snake–2013.

© 2022 Zhang C et al. *JAMA Network Open.*

eReferences.

1. Lee DS, Card D. Regression discontinuity inference with specification error. *Journal of Econometrics*. 2008 Feb 1;142(2):655–74.

© 2022 Zhang C et al. *JAMA Network Open.*
